# Associations of actigraphic sleep parameters with adipokines in older adults in the Baltimore longitudinal study of aging

**DOI:** 10.1093/sleepadvances/zpag082

**Published:** 2026-07-23

**Authors:** Zhikui Wei, Yiwei Yue, Jill A Rabinowitz, Marc Kaizi-Lutu, Mark N Wu, Chee W Chia, Toshiko Tanaka, Eleanor M Simonsick, Luigi Ferrucci, Adam P Spira

**Affiliations:** Department of Neurology and Jefferson Sleep Disorders Center, Thomas Jefferson University Hospital, Philadelphia, PA, United States; Department of Mental Health, Johns Hopkins Bloomberg School of Public Health, Baltimore, MD, United States; Department of Psychiatry, Robert Wood Johnson Medical School, Rutgers University, Piscataway, NJ, United States; Department of Mental Health, Johns Hopkins Bloomberg School of Public Health, Baltimore, MD, United States; Departments of Neurology and Neuroscience, Johns Hopkins University School of Medicine, Baltimore, MD, United States; Intramural Research Program, National Institute on Aging, National Institutes of Health, Baltimore, MD, United States; Intramural Research Program, National Institute on Aging, National Institutes of Health, Baltimore, MD, United States; Intramural Research Program, National Institute on Aging, National Institutes of Health, Baltimore, MD, United States; Intramural Research Program, National Institute on Aging, National Institutes of Health, Baltimore, MD, United States; Department of Neurology and Jefferson Sleep Disorders Center, Thomas Jefferson University Hospital, Philadelphia, PA, United States; Department of Psychiatry and Behavioral Sciences, Johns Hopkins University School of Medicine, Baltimore, MD, United States; Center on Aging and Health, Johns Hopkins University, Baltimore, MD, United States

**Keywords:** adipokines, actigraphy, sleep disturbance, metabolic dysfunction, older adults

## Abstract

**Study Objectives:**

Sleep and adipokines are important regulators of cardiometabolic health, yet their relationship in older adults remains poorly understood. We examined cross-sectional and longitudinal associations of actigraphic sleep parameters with adipokine levels among older adults and explored sex and central obesity as potential moderators.

**Materials and Methods:**

We studied 350 participants aged ≥60 years (mean = 74.8 ± 8.3 years, 48.9% females, 19.4% black) from the Baltimore Longitudinal Study of Aging who completed 6.5 ± 1.1 nights of wrist actigraphy and provided fasting plasma for adipokine measurements. Predictors were total sleep time (TST), sleep onset latency, wake after sleep onset, sleep efficiency, and average wake bout length (AWBL); outcomes were adiponectin and leptin levels.

**Results:**

At baseline, longer AWBL was associated with lower adiponectin (*B* = −2.02, 95% CI [−3.61, −0.42], *p* = .013) and leptin (*B* = −3.14, [−5.25, −1.03], *p* = .004) in fully adjusted models. Longer TST, modeled continuously, predicted a greater increase in adiponectin levels over time (*B* = 0.26, [0.081, 0.44], *p* = .007), and short TST (<6 h) was linked to subsequent decline in adiponectin (*B* = −0.90, [−1.72, −0.079], *p* = .038). Sex and central obesity moderated the baseline AWBL–adiponectin associations, which were significant in females (*B* = −3.92, [−6.25, −1.59], *p* = .001) and those with lower central obesity (*B* = −6.98, [−10.43, −3.53], *p* < .001).

**Conclusions:**

Longer sleep duration and shorter wake bouts are linked to more favorable adipokine profiles in older adults, especially in females and those with lower central obesity. Sleep may exert cardiometabolic effects through adipokines in older adults.

**Statement of Significance**

Sleep and adipokines play important roles in regulating cardiometabolic health, yet how objectively measured sleep parameters relate to adipokines in older adults remains unclear. This study identifies novel associations of longer sleep duration and more consolidated sleep with more favorable leptin and adiponectin patterns, suggesting a potential biological pathway through which sleep influences metabolic health during aging. The moderating effects of sex and central obesity on sleep-adiponectin associations highlight the importance of considering individual metabolic context when evaluating the impact of sleep on metabolic health. Together, these findings advance the understanding of the links between sleep and adipokine regulation in older adults.

## Graphical Abstract

**Graphical Abstract:**
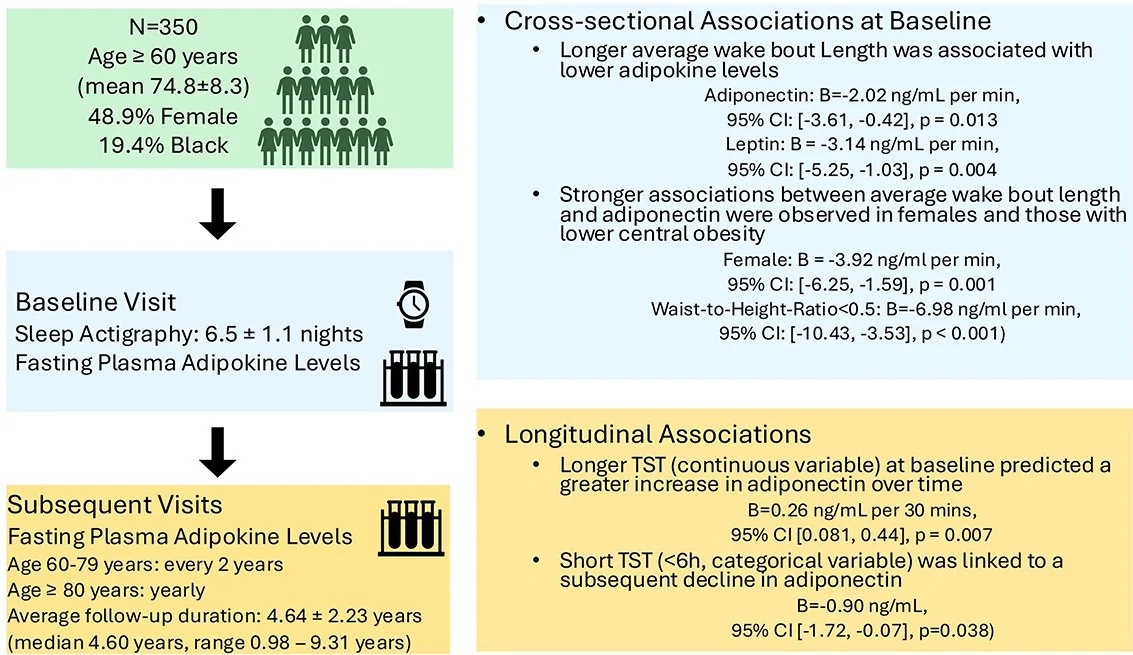

## Introduction

Sleep disturbances are common among older adults, with ~50% of persons aged 65 and older reporting difficulty initiating or maintaining sleep [1, 2]. Further, disturbed sleep is closely linked to cardiometabolic disease [3]. The biological mechanisms underlying the association between sleep disturbance and cardiometabolic disease are incompletely understood. One potential mechanism involves dysregulation of hormonal signals, such as adipokines [4].

Adipokines are a large group of protein or peptide hormones produced in adipose tissue that regulate whole-body metabolism through inter-organ crosstalk [5, 6]. Two representative adipokines, leptin and adiponectin, have been studied intensely in metabolic diseases due to their distinct biological functions [6]. Leptin suppresses appetite by acting on the satiety center of the hypothalamus, and adiponectin suppresses hepatic glucose production and increases fatty acid oxidation in skeletal muscle [6]. A higher adiponectin concentration is generally considered metabolically favorable due to its insulin-sensitizing and anti-inflammatory effects [7]. On the contrary, dysregulation of adipokines in obesity, such as higher leptin and lower adiponectin levels, has been linked to pathogenic processes in cardiometabolic disease, including insulin resistance, inflammation, and dyslipidemia [6, 7].

Accumulating evidence suggests that sleep disturbance could alter adipokine levels and contribute to systemic metabolic dysfunction in younger and middle-aged persons. For example, a study in the Wisconsin Sleep Cohort (aged 30–60 years) demonstrated that self-reported sleep duration of <8 h was associated with lower leptin and higher body mass index [8]. Similarly, results from the Quebec Family Study (aged 21–64 years) linked self-reported sleep duration <7–8 h to lower leptin levels and higher adiposity indices [9]. In contrast, findings from the Cleveland Family Study (mean age 44.5 years) linked shorter polysomnographic sleep duration to higher leptin levels [10]. Discrepancies in study findings could be due to differences in sleep measurement methods (i.e. self-report vs. objective measures) and heterogeneity in sample rates of obesity and diabetes [10].

Few studies have examined the associations between sleep and adipokines in older adults. This knowledge gap warrants further investigation, given the high prevalence of sleep disturbances and cardiometabolic disease in this population [1, 11]. Compared to younger adults, older adults have greater sleep fragmentation, more nighttime awakenings, and lower sleep efficiency [12]. These changes in sleep architecture have been linked to metabolic dysfunction [13, 14]. In addition, associations between sleep duration and adipokines observed in younger adults may not generalize to older adults due to aging-related changes in metabolism, hormonal regulation, and adipose tissue biology [15]. To better understand links between sleep and cardiometabolic health in older adults, we examined the cross-sectional and longitudinal associations of actigraphic sleep parameters with adipokine levels in older adults (≥60 years). We hypothesized that shorter sleep duration, longer sleep onset latency, lower sleep efficiency, and greater sleep fragmentation would be associated with lower baseline adiponectin and leptin levels and with greater longitudinal declines in these adipokine levels in older adults.

Central obesity, characterized by increased visceral adipose tissue, is an important marker of cardiometabolic risk and is associated with greater morbidity and mortality from cardiometabolic disease [16]. Visceral adipose tissue is a primary source of adipokines and exhibits a more pro-inflammatory adipokine expression profile (e.g. higher leptin and lower adiponectin expression) [6, 7]. Therefore, differences in visceral adiposity may modify the relationship between sleep and circulating adipokine levels. Sex strongly influences visceral adipose tissue accumulation, with men generally exhibiting greater visceral adipose tissue than women, particularly at older ages [17]. Accordingly, we explored sex and central obesity as moderators of sleep-adipokine associations in older adults. Waist-to-height ratio (WHtR) was used as an indicator of central obesity due to its superior ability to predict cardiovascular risk and its well-defined thresholds compared with other anthropometric parameters [18].

## Materials and methods

We studied participants from the Baltimore Longitudinal Study of Aging (BLSA), an ongoing, continuously enrolling community-based observational study aimed at identifying factors associated with cognitive and functional declines that can occur with aging [19]. The overall design and inclusion and exclusion criteria of the study were previously reported [20–23]. Briefly, participants were eligible to enroll in the BLSA if they were ≥20 years old and healthy (e.g. no history of major psychiatric, cognitive, or physical health limitations or illnesses, except for controlled hypertension or cancer within the past 10 years) [21–23]. To be eligible for inclusion in the current study, we required participants to be aged ≥60 years and to have available actigraphy and adipokine data. After enrollment in BLSA, participants are typically followed for life or until dementia diagnosis [20]. Participants aged <60 years are scheduled for assessments every 4 years [20–23]. Individuals aged 60–79 are scheduled for assessments every 2 years, and participants ≥80 years of age are scheduled for annual visits [20–23]. The current study was conducted within the BLSA cohort between 2012 and 2022 to evaluate associations between sleep parameters and adipokines. It was a hypothesis-driven analysis of existing cohort data. The current study sample consisted of participants aged ≥60 years who had wrist actigraphy at baseline and adipokine measurements (adiponectin and leptin) from baseline and, when available, at follow-up visits. We studied participants aged ≥60 years to capture an aging population while maintaining an adequate sample size and statistical power. The study “baseline” for each participant was defined as the first study visit at which they had both actigraphic and adipokine data. Participants completed, on average, 2.82 study visits (median: 3; range: 1–8), occurring approximately every 2 years (median: 1.99, interquartile range: 1.19, range: 0.87–7.67) between 2012 and 2022. The BLSA study protocol was reviewed by the National Institutes of Health IRB, and participants provided written informed consent at each study visit.

### Wrist actigraphy

Sleep parameters in this study were assessed using wrist actigraphy, which provides estimates of habitual sleep–wake patterns over multiple days in participants’ natural sleep environment. Some parameters, such as sleep onset latency and sleep efficiency, are informed by event markers and sleep diaries and therefore are not fully independent of participant input. However, actigraphy allows quantification of sleep characteristics that are difficult to capture accurately by self-report, such as sleep fragmentation, which increases with age and is particularly relevant when evaluating sleep in older adults. Actigraphy data were collected at a participant-specific baseline visit from 2012 to 2019. Participants were asked to wear a wrist actigraph (Actiwatch-2, Philips Respironics, Bend, OR) on their non-dominant wrist for 7 consecutive 24-h periods. Actigraphs were configured to collect data using the default settings of 10 immobile minutes to begin the sleep interval and the “medium” setting of 40 activity counts as a sensitivity threshold. Participants were instructed to press an event marker at “lights out” and upon final awakening. Concurrent sleep diaries were completed to record bedtimes, wake times, naps, and periods of device removal. Rest intervals (time in bed) were defined using a combination of event markers and sleep diary entries, with visual inspection and manual adjustment when discrepancies were present. Periods of non-wear were identified from diaries and excluded from analyses. Actigraphy data were processed using Actiware software v6.0.9 (Philips-Respironics), which applies a validated algorithm to classify sleep and wake. Actigraphy data were scored independently by two research staff members using Spira lab’s standard operating procedures [24]. Discrepancies in scoring were resolved by consensus.

We calculated the mean total sleep time (TST) (total minutes spent asleep while in bed), sleep onset latency (number of minutes from “lights out” until sleep onset), sleep efficiency (percentage (%) of time in bed spent asleep); wake after sleep onset (total minutes spent awake after sleep onset); and average wake bout length (total number of minutes awake divided by number of wake bouts) as previously reported [25]. Each sleep parameter was averaged across the available nights (up to 7) to obtain a stable estimate of habitual sleep and treated as a baseline exposure variable.

### Assessment of adipokines (i.e. adiponectin and leptin)

Blood samples were collected between 7:00 and 9:30 am following an overnight fast [26]. They were processed immediately and subsequently stored at a temperature of −80 °C. Assays were conducted at the Laboratory of Clinical Investigation and its Diabetes Section, located at the National Institute on Aging in Baltimore, MD, USA. Plasma leptin was measured using an enzyme-linked immunosorbent assay (ELISA) from Linco Research, Inc, in St. Charles, MO, USA [26]. Plasma adiponectin was determined by a radioimmunoassay (Linco Research) [26]. Leptin and adiponectin concentrations were expressed in ng/mL and analyzed as continuous variables. Plasma adipokine levels were measured at each study visit, which occurred approximately every 2 years (median: 1.99, interquartile range: 1.19, range: 0.87–7.67) between 2012 and 2022 for the study sample.

### Covariates

Demographics (age, sex, race, and education) were obtained via interview. Race was self-reported and categorized as White, Black/African American, and other (which includes many different racial and ethnic groups), and subsequently dichotomized as Black/African American and White/other due to the small number of participants identifying as “other.” Education was categorized as: (1) some college or less; (2) college graduate; and (3) graduate education or higher. Waist circumference and height were measured using standardized protocols in triplicate, with additional measurements obtained if values were outside expected ranges at each visit. WHtR was calculated by dividing waist circumference by height and binarized by applying the at-risk cutoff point ≥0.50 [27, 28], as WHtR greater than 0.50 is linked to increased mortality risk from cardiometabolic disease [29]. Comorbidities, including hypertension or diabetes (type 2), were obtained via interview.

### Statistical analyses

We used *t*-tests and chi-squared tests to assess differences in sleep parameters, adipokines, and other covariates by sex and WHtR categories. All continuous covariates were mean-centered. TST was mean-centered and scaled per 30 min. Sleep onset latency and wake after sleep onset were mean-centered and scaled per 10 min. Sleep efficiency was mean-centered and scaled per 10%.

For each sleep predictor, we fit three cross-sectional and longitudinal models. Model 1 adjusted for age, sex, race, and education. Model 2 added WHtR. Model 3 further included diabetes and hypertension. Cross-sectional analyses used linear regression to assess associations between each sleep parameter and adipokine levels at baseline. Longitudinal analyses used linear mixed effects models with each sleep parameter, time in follow-up years, covariates, and interaction terms with time to test whether baseline sleep predicted changes in adipokine levels. Follow-up time was modeled linearly to provide a readily interpretable estimate of longitudinal change aligned with the study objective. Both cross-sectional and longitudinal analyses were performed to make full use of the available data and provide a more comprehensive understanding of the sleep–adipokine relationship in an aging cohort. We tested moderation by adding sleep parameter × moderator (sex or WHtR) interactions in the cross-sectional models and sleep parameter × moderator × time interactions in the longitudinal models. When interactions were statistically significant (*p* < .05), estimates of the associations between sleep parameters and adipokines were presented for each sex or WHtR subgroup. We then performed post hoc Wald tests to evaluate between-group differences in slopes across sex and WHtR subgroups.

Four participants had fewer than three nights of actigraphy. Sensitivity analyses excluding these participants were performed for significant findings. Estimates, confidence intervals, and *p*-values remained virtually unchanged; therefore, results are presented for the full analytic sample (*n* = 350). Sensitivity analyses were also performed by excluding two participants with two nights of no detected sleep and one participant with one sleep variable (average wake bout length) that could not be calculated for one night. As results were unchanged (data not shown), all participants were retained in the final analyses. All cross-sectional and longitudinal analyses were conducted using RStudio version 2024.04.2 + 764. Generative AI was used to assist with language editing and with debugging and polishing R code.

## Results

Among the 350 participants, the mean age was 74.8 ± 8.3 (range 60–96) years (Table 1), 171 (48.9%) were female, and 68 (19.4%) reported their race as black. Participants completed an average of 6.5 ± 1.1 nights of wrist actigraphy (Median = 7 nights; range 1–7 nights). Sixty-five participants (18.6%) had incomplete actigraphy (<7 nights), with an average of 2.5 ± 1.2 (median = 2; range 1–6) missing nights. Of the 350 participants, 269 (77%) had follow-up visits post-baseline visit with an average follow-up duration of 4.64 ± 2.23 years (median 4.60 years, range 0.98–9.31 years). Mean age, sex, race, education level, and baseline sleep parameters of participants with more than 1 study visit were similar to those who only had a baseline visit (*N* = 81, 23% of total sample, data not shown), except that average-wake bout length was slightly longer in those with follow-up data compared to those who did not (1.92 ± 0.83 min vs. 1.60 ± 0.75 min, *p* = .001).

**Table 1:** Descriptive statistics for the participants stratified by sex and waist-to-height ratio

Characteristics	Total	Male	Female	*p*-value	Waist-to-height ratio < 0.5	Waist-to-height ratio ≥ 0.5	*p*-value
	*N* = 350	*N* = 179	*N* = 171		*N* = 110	*N* = 239	
**Age, *M* (*SD*)**	74.81 (8.31)	76.23 (8.36)	73.32 (8.02)	**<.001**	74.77 (8.26)	74.85 (8.36)	.94
**Sex, *N* (%)**							**<.001**
Male	179 (51.1%)	-	-	**-**	35 (31.8%)	143 (59.8%)	
Female	171 (48.9%)	-	-	**-**	75 (68.2%)	96 (40.2%)	
**Race, *N* (%)**				**.036**			.48
White and other	282 (80.6%)	152 (84.9%)	130 (76.0%)		91 (82.7%)	190 (79.5%)	
Black	68 (19.4%)	27 (15.1%)	41 (24.0%)		19 (17.3%)	49 (20.5%)	
**Education, *N* (%)**				.47			.48
Some college or less	42 (12.0%)	20 (11.2%)	22 (12.9%)		11 (10.0%)	31 (13.0%)	
College graduate	68 (19.4%)	31 (17.3%)	37 (21.6%)		25 (22.7%)	43 (18.0%)	
Graduate education and above	240 (68.6%)	128 (71.5%)	112 (65.5%)		74 (67.3%)	165 (69.0%)	
**Waist-to-height ratio**	0.54 (0.06)	0.56 (0.06)	0.52 (0.07)	**<.001**	**-**	**-**	**-**
**Waist-to-height ratio ≥ 0.5**				**<.001**	**-**	**-**	**-**
No	110 (31.4%)	35 (19.6%)	75 (43.9%)		**-**	**-**	**-**
Yes	239 (68.3%)	143 (79.9%)	96 (56.1%)		**-**	**-**	**-**
Missing	1 (0.3%)	1 (0.6%)	0		**-**	**-**	**-**
**Hypertension, *N* (%)**				.19			.16
No	181 (51.7%)	86 (48.0%)	95 (55.6%)		65 (59.1%)	116 (48.5%)	
Yes	167 (47.7%)	91 (50.8%)	76 (44.4%)		45 (40.9%)	122 (51.0%)	
Missing	2 (0.6%)	2 (1.1%)	0		0	1 (0.4%)	
**Diabetes, *N* (%)**				.24			.23
No	296 (84.6%)	147 (82.1%)	149 (87.1%)		97 (88.2%)	199 (83.3%)	
Yes	53 (15.1%)	31 (17.3%)	22 (12.9%)		13 (11.8%)	40 (16.7%)	
Missing	1 (0.3%)	1 (0.6%)	0		-	-	**-**
**Total sleep time (min), *M* (*SD*)**	403.25 (62.42)	393.60 (63.85)	413.36 (59.40)	**.003**	415.74 (50.60)	397.80 (66.47)	**.012**
**Sleep onset latency (min), *M* (*SD*)**	13.15 (13.08)	13.90 (13.82)	12.37 (12.26)	.28	10.72 (9.88)	14.23 (14.22)	**.020**
**Sleep efficiency (%), *M* (*SD*)**	83.46 (7.44)	82.36 (7.82)	84.61 (6.85)	**.005**	84.82 (5.49)	82.84 (8.13)	**.021**
**Wake after sleep onset (min), *M* (*SD*)**	47.83 (21.65)	48.86 (21.92)	46.74 (21.37)	.36	48.55 (21.68)	47.52 (21.71)	.68
**Average wake bout length (min), *M* (*SD*)**	1.84 (0.82)	1.80 (0.86)	1.89 (0.78)	.27	1.92 (0.66)	1.81 (0.89)	.26
**Adiponectin (ng/mL), *M* (*SD*)**	15.46 (12.38)	13.33 (10.58)	17.70 (13.70)	**<.001**	18.21 (14.68)	14.24 (10.98)	**.005**
**Leptin (ng/mL), *M* (*SD*)**	17.80 (17.36)	12.79 (12.69)	23.04 (19.89)	**<.001**	14.41 (14.69)	19.41 (18.29)	**.012**

Bolded values indicate statistical significance.

There were significant sex differences in participant characteristics (Table 1). Males were older and had higher WHtR than females, and a higher proportion of females than males identified as black. Females also had longer TST, higher sleep efficiency, and higher levels of adiponectin and leptin compared to males.

Compared to participants with WHtR ≥0.5, those with WHtR <0.5 had a lower proportion of male participants, longer TST, shorter sleep onset latency, and higher sleep efficiency.

### Cross-sectional and longitudinal associations between sleep parameters and adipokines

In all cross-sectional models, longer average wake bout length was significantly associated with lower adiponectin and lower leptin at baseline (Table 2: Model 3, adiponectin *B* = −2.02 ng/mL per minute, 95% CI [−3.61, −0.42], *p* = .013; leptin *B* = −3.14 ng/mL per minute, 95% CI [−5.25, −1.03], *p* = .004). No other sleep parameters were associated with adiponectin or leptin levels at baseline.

**Table 2:** Cross-sectional associations of sleep parameters with adipokines at baseline, reported as *B* (95% CI)

Sleep parameters	Model 1		Model 2		Model 3	
	Adiponectin	Leptin	Adiponectin	Leptin	Adiponectin	Leptin
Total sleep time	−0.45 (−1.09, 0.18)	−0.18 (−1.04, 0.68)	−0.52 (−1.16, 0.12)	−0.04 (−0.88, 0.81)	−0.60 (−1.24, 0.04)	−0.10 (−0.96, 0.75)
Sleep onset latency	0.30 (−0.70, 1.29)	0.69 (−0.64, 2.02)	0.42 (−0.58, 1.41)	0.41 (−0.90, 1.72)	0.50 (−0.49, 1.50)	0.37 (−0.95, 1.69)
Sleep efficiency	−0.03 (−1.81, 1.75)	−0.10 (−2.49, 2.29)	−0.17 (−1.95, 1.61)	0.29 (−2.06, 2.63)	−0.53 (−2.32, 1.27)	0.19 (−2.19, 2.58)
Wake after sleep onset	−0.54 (−1.13, 0.06)	−0.39 (−1.19, 0.41)	−0.56 (−1.15, 0.03)	−0.34 (−1.12, 0.45)	−0.46 (−1.06, 0.14)	−0.35 (−1.14, 0.45)
Average wake bout length	−2.19^**^ **(**−**3.77,** −**0.61)**	−3.28^**^ **(**−**5.40,** −**1.16)**	−2.25^**^ **(**−**3.82,** −**0.68)**	−3.12^**^ **(**−**5.20,** −**1.04)**	−2.02^*^ **(**−**3.61,** −**0.42)**	−3.14^**^ **(**−**5.25,** −**1.03)**

*p*-value <.05^*^; *p*-value <.01^**^.

Model 1 adjusted for age, sex, race, and education, Model 2 further adjusted for waist-to-height ratio, and Model 3 further adjusted for diabetes and hypertension. Bolded values indicate statistical significance.

In longitudinal analyses, longer TST was associated with a greater increase in adiponectin levels across all models (Table 3). For example, in Model 3, each 30-min increase in TST above the sample mean was associated with a 0.26 ng/mL increase in adiponectin (*B* = 0.26, 95% CI [0.081, 0.44], *p* = .007). No significant association was found between TST and changes in leptin levels. No other sleep parameters were significantly associated with changes in adipokine levels.

**Table 3:** Longitudinal associations between sleep parameters and adipokines, reported as *B* (95% CI)

Sleep parameters	Model 1		Model 2		Model 3	
	Adiponectin	Leptin	Adiponectin	Leptin	Adiponectin	Leptin
Total sleep time	**0.17** ^*^ **(0.021, 0.32)**	−0.076 (−0.37, 0.22)	**0.17** ^*^ **(0.020, 0.32)**	−0.075 (−0.38, 0.23)	**0.26** ^*^ **(0.081, 0.44)**	−0.089 (−0.41, 0.23)
Sleep onset latency	−0.19 (−0.48, 0.10)	0.16 (−0.44, 0.77)	−0.19 (−0.48, 0.10)	0.22 (−0.39, 0.83)	−0.26 (−0.62, 0.095)	0.21 (−0.40, 0.82)
Sleep efficiency	0.17 (−0.23, 0.57)	−0.29 (−1.11, 0.52)	0.17 (−0.22, 0.57)	−0.38 (−1.23, 0.45)	0.39 (−0.11, 0.88)	−0.36 (−1.21, 0.49)
Wake after sleep onset	0.046 (−0.084, 0.18)	0.028 (−0.23, 0.29)	0.046 (−0.083, 0.18)	0.040 (−0.22, 0.30)	−0.018 (−0.16, 0.13)	0.076 (−0.18, 0.33)
Average wake bout length	0.35 (−0.006, 0.70)	0.22 (−0.52, 0.97)	0.36 (0.004, 0.71)	0.19 (−0.56, 0.96)	0.093 (−0.35, 0.54)	0.39 (−0.36, 1.17)

Values represent *B* estimates for interaction terms between sleep parameters and time, indicating longitudinal change

*p*-value <.05^*^; *p*-value <.01^**^.

Model 1 adjusted for age, sex, race, and education as well as their interactions with time, Model 2 further adjusted for waist-to-height ratio and its interaction with time, and Model 3 further adjusted for diabetes and hypertension and their interactions with time. Bolded values indicate statistical significance.

We also examined whether short (<6 h) or long (>9 h) versus intermediate (6–9 h) TST predicted changes in adiponectin or leptin over time (Table 4). Categorical sleep duration was included because of the recognized “J-shaped” relationship between sleep duration and metabolic outcomes, in which both short and long sleep durations were associated with adverse metabolic outcomes in older adults [13]. Compared to participants with intermediate sleep time, those with short TST showed a decline in adiponectin (*B* = **−**0.90 ng/mL, 95% CI [−1.72, −0.079], *p* = .038) in Model 3; no effect was observed for leptin. Long TST was not significantly associated with changes in either adiponectin or leptin.

**Table 4:** Longitudinal associations between short vs. long total sleep time and adipokines, reported as *B* (95% CI)

Categorical sleep duration	Model 1		Model 2		Model 3	
	Adiponectin	Leptin	Adiponectin	Leptin	Adiponectin	Leptin
Intermediate total sleep time (6–9 h)	Reference	Reference	Reference	Reference	Reference	Reference
Short total sleep time (<6 h)	−0.64 (−1.32, 0.045)	0.57 (−0.79, 1.91)	−0.66 (−1.34, 0.024)	0.50 (−0.90, 1.86)	**−0.90** ^*^ **(−1.72, −0.079)**	−0.007 (−1.41, 1.38)
Long total sleep time (>9 h)	−1.31 (−5.05, 2.38)	−1.38 (−8.47, 5.63)	−1.49 (−5.20, 2.16)	−1.48 (−8.61, 5.54)	−1.60 (−5.74, 2.47)	−0.50 (−7.69, 6.57)

Values represent *B* estimates for interaction terms between sleep parameters and time, indicating longitudinal change.

*p*-value < .05^*^.

Model 1 adjusted for age, sex, race, and education as well as their interactions with time, Model 2 further adjusted for waist-to-height ratio and its interaction with time, and Model 3 further adjusted for diabetes and hypertension and their interactions with time. Bolded values indicate statistical significance.

### Interactions of sleep with sex and WHtR in cross-sectional analysis

Sex interacted with average wake bout length with respect to adiponectin at baseline (Model 3, *p* for interaction = .029; Table 5). Model-derived sex-specific estimates showed that greater average wake bout length was associated with lower levels of adiponectin at baseline in females (*B* = −3.92 ng/mL per minute, 95% CI [−6.25, −1.59], *p* = .001). The magnitude of effect was much lower and non-significant among males (*B* = −0.44 ng/mL per minute, 95% CI [−2.57, 1.68], *p* = .683). Post hoc Wald testing confirmed that the slope was significantly more negative in females than in males (*B* for difference in slopes [female − male] = −3.48 ng/mL per minute, 95% CI [−6.61, −0.35], *p* = .029). There were no longitudinal interactions of sex and sleep parameters with adipokines.

**Table 5:** Interactions of average wake bout length and sex in predicting baseline adiponectin, reported as *B* (95% CI)

**Model 1**				
Sleep parameter	Female	Male	Interaction *p*-value	Post hoc Wald test
Average wake bout length	**−4.14 (−6.46, −1.81)** ^**^	−0.58 (−2.69, 1.53)	**.026** ^*^	**−3.55 (−6.68, −0.43)** ^*^
**Model 2**				
**Sleep parameter**	**Female**	**Male**	**Interaction *p*-value**	**Post hoc Wald test**
Average wake bout length	**−4.03 (−6.35, −1.71)** ^**^	−0.78 (−2.89, 1.34)	**.042** ^*^	**−3.25 (−6.37, −0.13)** ^*^
**Model 3**				
**Sleep parameter**	**Female**	**Male**	**Interaction *p*-value**	**Post hoc Wald test**
Average wake bout length	**−3.92 (−6.25, −1.59)** ^**^	−0.44 (−2.57, 1.68)	**.029** ^*^	**−3.48 (−6.61, −0.35)** ^*^

Model 1 adjusted for age, sex, race, and education, Model 2 further adjusted for waist-to-height ratio, and Model 3 further adjusted for diabetes and hypertension.

^*^ *p*-value <.05; ^**^p-value <.01.

The post hoc Ward test represented the between-sex difference in slopes (female−male). Bolded values indicate statistical significance.

WHtR interacted with average wake bout length and baseline adiponectin (Model 2, *p* for interaction = .002, Table 6). Among participants with WHtR <0.5, longer average wake bout length was associated with lower levels of adiponectin (*B* = −6.98 ng/mL per min, 95% CI [−10.43, −3.53], *p* < .001), whereas the association was non-significant in those with WHtR ≥0.5 (Table 6). Post hoc Wald testing confirmed that the slope was significantly more negative in participants with WHtR <0.5 than in those with WHtR ≥0.5 (*B* for difference in slopes [WHtR < 0.5 − WHtR ≥ 0.5] = −6.19 ng/mL per min, *p* = .002). In longitudinal models, the negative association between average wake bout length and adiponectin in the WHtR <0.5 group at baseline persisted over follow-up with no significant interactions between the sleep parameter and time, while no significant WHtR interactions were observed for other sleep parameters with adiponectin or leptin. However, because non-linear trajectories were not examined, more complex time-dependent interactions cannot be excluded.

**Table 6:** Interactions of average wake bout length and waist-to-height ratio (WHtR) in predicting baseline adiponectin, reported as *B* (95% CI)

**Model 1**				
Sleep parameter	WHtR < 0.5	WHtR ≥ 0.5	Interaction *p*-value	Post hoc Wald test
Average wake bout length	**−7.17 (−10.62, −3.72)** ^**^	−1.03 (−2.76, 0.70)	**.002** ^**^	**−6.14 (−9.98, −2.30)** ^**^
**Model 2**				
**Sleep parameter**	**WHtR < 0.5**	**WHtR ≥ 0.5**	**Interaction *p*-value**	**Post hoc Wald test**
Average wake bout length	**−6.98 (−10.43, −3.53)** ^**^	−0.79 (−2.53, 0.96)	**.002** ^**^	**−6.19 (−10.01, −2.37)** ^**^

Model 1 adjusted for age, sex, race, education, and waist-to-height ratio, and Model 2 further adjusted for diabetes and hypertension.

^*^ *p*-value <.05; ^**^p-value <.01.

The post hoc Wald test represented the between-group difference in slopes (WHtR < 0.5 − WHtR ≥ 0.5). Bolded values indicate statistical significance.

## Discussion

We investigated the cross-sectional and longitudinal associations between objectively measured sleep parameters and adipokine levels in older adults. In cross-sectional analyses, we found significant inverse associations between average wake bout length and adiponectin and leptin levels at baseline. Longitudinally, longer TST at baseline predicted positive changes in adiponectin over time. We also identified sex and central obesity as moderators of the cross-sectional association between average wake bout length and adiponectin levels at baseline. These findings highlighted the significant associations between sleep disturbance and adipokines in older adults.

To our knowledge, this is the first study that demonstrated an inverse association between average wake bout length and adiponectin or leptin in older adults. Compared to sleep of younger adults, older adults’ sleep is characterized by greater fragmentation, including more frequent arousals and longer nocturnal awakenings [12]. Average wake bout length was included as a measure of sleep fragmentation, reflecting the average duration of wake bouts and the subject’s ability to return to sleep. Adiponectin and leptin play central roles in the regulation of metabolism and energy balance [6, 7]. Lower levels of adiponectin are generally associated with insulin resistance, while lower levels of leptin can indicate decreased satiety and increased caloric intake [6, 7]. Therefore, our findings suggest that disrupted sleep patterns or greater difficulty returning to sleep characterized by longer wake bouts are associated with altered secretion and regulation of these adipokines, potentially exacerbating metabolic dysregulation such as insulin resistance and excess caloric intake. Further research is needed to confirm whether average bout length is associated with changes in adipokines over time, which may help identify pathways that could mitigate the adverse effects of nocturnal awakenings or enhance the protective effects of sleep on cardiometabolic health among older adults.

We also identified WHtR as a significant moderator of the association between average wake bout length and adiponectin at baseline, such that the inverse association was significant only in those with lower WHtR. Central obesity is well known to be associated with sleep disturbances [30]. This aligns with our observations that participants with WHtR ≥0.5 had shorter TST, lower sleep efficiency, and longer sleep onset latency (Table 1). Further, central obesity also regulates adipokine biology by contributing to a low-grade inflammatory milieu in which pro-inflammatory cytokines (e.g. TNF-α, IL-6) are upregulated, and adiponectin is downregulated [31]. Consistent with this understanding, participants with higher WHtR have lower adiponectin levels in our study sample. Thus, the inverse association between average wake bout length and adiponectin observed in participants with lower WHtR may have been masked in those with higher WHtR by the overriding effects of central obesity.

In line with this interpretation, the inverse relationship between average wake bout length and adiponectin levels at baseline in females but not in males may be due to significant sex-specific differences in adipose tissue biology with aging [32]. Compared to older women, older men generally exhibit greater central obesity with more visceral adipose tissue [32]. They also tend to have lower adiponectin and leptin levels [33]. In contrast, older women accumulate more subcutaneous fat, which is less metabolically adverse [17]. These changes are partially attributable to sex hormones. It is thought that visceral adipose tissue may be primarily regulated by testosterone, whereas female hormones, i.e. estrogen and progesterone, promote subcutaneous adiposity [32]. In our cohort, males had greater central obesity, reflected by higher WHtR, along with lower adiponectin and leptin levels than females (Table 1). These differences suggest that central obesity may play a more dominant role in adipokine dysregulation among male participants in our study, potentially attenuating or obscuring the association between sleep fragmentation and adipokines observed in females. Future studies involving older men and women with low central obesity are needed to clarify these relationships. Nevertheless, our findings highlight the importance of considering sex differences when addressing sleep and metabolic health in older individuals [34].

We found that longer TST predicted a greater increase in adiponectin levels, and short sleep duration (<6 h) was associated with a decline in adiponectin levels in older adults. To our knowledge, this is the first study that demonstrated a positive longitudinal association between TST and changes in adiponectin levels in adults, although similar associations have been observed in cross-sectional studies. For example, a study showed a positive cross-sectional association between sleep duration and adiponectin levels in healthy Japanese men, with a stronger association in those aged >55 years [35]. Our results provide longitudinal support for a role of sleep duration in modulating adiponectin changes, extending prior cross-sectional observations. The mechanisms linking sleep duration to changes in adiponectin levels are unclear. Some evidence suggests that adrenergic tone can modulate adiponectin expression and secretion. A prior basic science study showed that β-adrenergic agonists inhibited adiponectin production and secretion in adipose tissue [36]. In addition, adrenergic tone decreases during sleep, and sleep loss increases adrenergic tone by activating the sympathetic nervous system [4, 37]. These observations raise the possibility that short sleep duration may reduce adiponectin expression and secretion through increased adrenergic activity.

In this study, short sleep duration was defined as TST<6 h, intermediate sleep duration as TST 6–9 h, and long sleep duration as TST >9 h. Similar definitions of categorical sleep duration have been used in epidemiologic studies of older adults [38, 39]. These definitions differ somewhat from the 2015 American Academy of Sleep Medicine (AASM) and Sleep Research Society (SRS) consensus recommendation of 7–9 h of sleep for adults aged 18–60 years [40]. As our study population consists of older adults aged ≥60 years, the AASM/SRS consensus recommendation may not fully reflect the optimal sleep duration in our study sample. For example, age-specific recommendations from the National Sleep Foundation for adults aged ≥65 years identify 7–8 h as the recommended sleep duration, while 5–6 h and 9 h “may be appropriate” [41]. Given these recommendations, we elected to use a more inclusive reference group for intermediate sleep duration (6–9 h), and <6 h and >9 h as short and long sleep duration categories, respectively.

The lack of cross-sectional association between TST and adipokine levels at baseline in our study was unexpected, given the demonstrated associations from prior studies [8–10]. There are two possible explanations for these findings. First, our sample size is smaller compared to prior studies [8–10]. It is possible that with a larger number of participants, we may be able to observe a significant association between TST and adipokine levels at baseline. Second, age-related changes in sleep could also be a contributing factor. Older adults have greater variability in their TST than younger adults [42]. Participants in the present study are significantly older, with a mean age of 74.8 years, than those in prior studies, and may have greater variability in their TST, which could have affected our ability to detect a cross-sectional association of TST and adipokines at baseline.

This study has several limitations. While the sample size in this research was adequate to examine primary associations between sleep and adipokines in older adults, it is smaller than prior studies performed on sleep and adipokines in younger and middle-aged adults [10], suggesting a potential need for larger sample sizes in future studies to investigate associations of different sleep parameters and their associations with adipokines. In this study, we included multiple actigraphic sleep parameters representing complementary sleep dimensions derived from the same actigraphy recordings and were selected based on prespecified hypotheses. Therefore, formal correction for multiple comparisons was not applied. However, given the number of analyses performed, there is a potential for increased Type I error, and findings should be validated by future studies. In addition, although moderator analyses by sex and central obesity were based on biological plausibility and specified a priori, the study may be underpowered to detect moderator effects. The moderator analyses are therefore considered exploratory. The actigraphic sleep parameters were assessed at baseline only and were not repeated alongside adipokine measurements over time. This may represent a missed opportunity to identify significant longitudinal associations between some sleep parameters and adipokines. The use of actigraphy to measure sleep patterns in this study does not account for sleep-related breathing disorders, such as obstructive sleep apnea, an important sleep disorder that can influence the cardiometabolic health of older adults. Older adults are at increased risk for sleep-related breathing disorders compared with younger and middle-aged adults [43]. As such, the potential impact of sleep-disordered breathing on the observed associations cannot be determined or excluded. Some actigraphic parameters, such as sleep onset latency and sleep efficiency, are informed by event markers and sleep diaries and, therefore, are not fully independent of participant input and are more vulnerable to measurement error. These limitations highlight the need for future research that incorporates more comprehensive sleep evaluations, such as polysomnography, to assess the sleep and metabolic health of older adults. Lastly, it is important to note that the participants in this study are, on average, highly educated and in relatively better health than many community-dwelling older adults, possibly limiting the generalizability of the findings to other community-dwelling older adults.

In conclusion, our study showed that longer average wake bout length was associated with lower levels of adiponectin and leptin in older adults at baseline, and longer TST was associated with a greater increase, whereas short sleep duration was associated with a decline in adiponectin levels over time. Stronger associations between average wake bout length and adiponectin were observed in females and in individuals with lower central obesity. Our results suggest that sleep fragmentation and short sleep duration are linked to adipokine dysregulation, whereas more continuous sleep and longer sleep duration are associated with more favorable adipokine profiles in older adults. These findings highlight the potential role of sleep in modulating adipokine biology in aging. Future studies are needed to determine whether these relationships are causal or reflect changes in metabolic health in older adults.

## Data Availability

Data are available through reasonable request.
